# Host miRNA orchestration of mitophagy pathways during bacterial infection: current advances and future directions

**DOI:** 10.3389/fmicb.2026.1941726

**Published:** 2026-09-24

**Authors:** Xin Hu, Wei Jiang, Xiangting Yuan, Qinqin Pu, Kelei Zhao, Ting Huang

**Affiliations:** 1 Clinical Medical College and Affiliated Hospital of Chengdu University, Chengdu University, Chengdu, China; 2 Antibiotics Innovation and Resistance Control Key Laboratory of Sichuan Province, School of Pharmacy, Chengdu University, Chengdu, China; 3 Department of Microbiology, University of Pennsylvania, Philadelphia, PA, United States

**Keywords:** bacterial pathogens, host defense, infectious diseases, microRNAs, mitophagy

## Abstract

Pathogenic bacterial infections continue to impose a substantial global health burden. MicroRNAs (miRNAs), a class of small non-coding RNAs, have emerged as critical post-transcriptional regulators of host innate immunity and inflammatory responses during bacterial pathogenesis. Accumulating evidence indicates that miRNAs dictate the outcomes of infection by orchestrating fundamental cellular processes, notably mitophagy—a selective autophagic pathway essential for mitochondrial quality control and cytosolic surveillance. While recent studies have begun to elucidate the molecular interplay between miRNA biogenesis and mitophagy regulation, the mechanistic crosstalk between miRNAs and mitophagy during bacterial infection remains fragmentary, and many reported miRNA-mitophagy regulatory relationships are derived from non-infectious disease models rather than bacterial infection settings. In this review, we synthesize current advances regarding miRNA-mediated modulation of core mitophagy machinery and associated signaling cascades in the context of bacterial infection. We further explore the pathophysiological implications of these regulatory networks and evaluate the therapeutic potential of targeting specific miRNA–mitophagy axes. This study provides mechanistic insights into how host miRNAs reprogram mitophagy to combat infection and the miRNA–mitophagy axis maybe as a promising dual-targeted therapeutic strategy for the development of host-directed therapies against bacterial diseases.

## 1 Introduction

As a powerhouse of cell, mitochondria are vital organelles of cell involved in different fundamental cell functions, from energy production (adenosine triphosphate, ATP) and fatty acid oxidation to modulating inflammation, proliferation, and cell survival/death (Tiku et al., 2020). Mitochondria derived from an endosymbiotic relationship between ancient eukaryotic cells and bacteria a billion years ago (Valt et al., 2026). These double-membrane-bounded organelles encode only 13 proteins by their own mitochondrial-DNA (mtDNA) and the rest 99% of mitochondrial-related proteins were encoded via nuclear genome (Richter-Dennerlein et al., 2026). Septic and damaged mitochondria lead to overproduction of reactive oxygen species (ROS) and the other oxidants, subsequently modifying the electron transport chain of cells (Tiku et al., 2020). Due to the important functions of mitochondria in the cell, mitochondrial dysfunctions cause various diseases, and are thus potential promising targets for controlling these diseases (Borcherding and Brestoff, 2023; Xie et al., 2025).

Selective mitochondrial autophagy (mitophagy), is also a conserved cellular self-digestion procedure in the elimination of mitochondrial dysfunction arising from damage or infection for maintaining cellular homeostasis (Gao et al., 2024; Ma et al., 2023). It is important to discriminate mitophagy from non-selective autophagy, which mediates the generalized turnover of cytoplasmic components without mitochondrial specificity (Singhal et al., 2023). In this process, damaged mitochondria are fused with autophagosomes, the double membrane structures, which are then transported to lysosomes for the cargoes’ degradation (Picca et al., 2023). If the damaged mitochondria are allowed to remain, the reactive oxygen species (ROS) will be increased and ATP synthesis will be decreased, which eventually leading to apoptosis of the cells (Saller et al., 2025). Two main categories of mitophagy have been widely investigated: PINK1/Parkin-mediated mitophagy and receptor-mediated mitophagy. In recent years, mitophagy has become a popular topic of research due to increasing evidence for the important role in several human pathologies, including neurodegenerative diseases, cardiovascular diseases, and cancer (D'Arcy, 2024). Notably, an increasing body of evidence has revealed that dysregulation of mitophagy seems to be accountable for the pathogenesis of bacterial infections, indicating the significant role of its precise control (Hajra et al., 2025; Verbeke et al., 2023a,b). Several mitochondrial-related proteins contribute to activation of mitophagy and phagolysosomal maturation in the process of microbial invasion (Hajra et al., 2025; Huang et al., 2020b; Verbeke et al., 2023b). In addition, mitophagy have a pivotal role in host resistance against bacterial infections, including the regulation of inflammasome activation and induction of bactericidal ROS (Huang et al., 2020b; Luo et al., 2023). Mitophagy exerts context-dependent dual functions during bacterial infection: physiological mitophagy can protect host cells by removing injured mitochondria and restraining excessive inflammatory responses; however, excessive mitophagy may trigger mitochondrial depletion and cell death, aggravating infection-associated tissue injury. Moreover, certain intracellular bacterial pathogens hijack host mitophagy pathways to construct an intracellular survival niche.

MicroRNAs (miRNAs), a well-known class of non-coding RNAs with less 22 nucleotides, could regulate gene expression via targeting specific mRNAs for translational repression or degradation at a post-transcriptional level (Mishra et al., 2020; Zhao et al., 2022). Mechanistically, functional miRNAs are stabilized by the miRNA-induced silencing complex (miRISC) and guide it to complementary sites in the 3′-untranslated region (3′-UTR) of target mRNAs. The imperfect or perfect complementarity binding sites subsequently lead to repression or degradation of target mRNA at the post-transcriptional levels (Abbas et al., 2023; Jeepipalli et al., 2025) (Figure 1). As the major regulators of gene expression, miRNAs have a curial role in numerous biological pathways involved in growth, homeostasis, and in response to disease progression (Tamaddon et al., 2025). In recent years, multitudinous studies have demonstrated the function and feedback mechanisms of miRNAs in both of the intracellular and extracellular organelles, which implying that these versatile miRNAs are potential diagnostic and therapeutic targets because of their role in human diseases (Huang et al., 2020a; Li, W. et al., 2026). A recent comprehensive review has systematically summarized the regulatory roles of miRNAs in host immune responses against major bacterial pathogens (Li, L. et al., 2026). With the development of genomics and bioinformatics tools including high-throughput sequencing technique and single cell RNA sequencing, microRNAs have been found to participate in the regulation of autophagy-related pathways such as mitophagy (Kong et al., 2021; Zhang et al., 2026).

**Figure 1:**
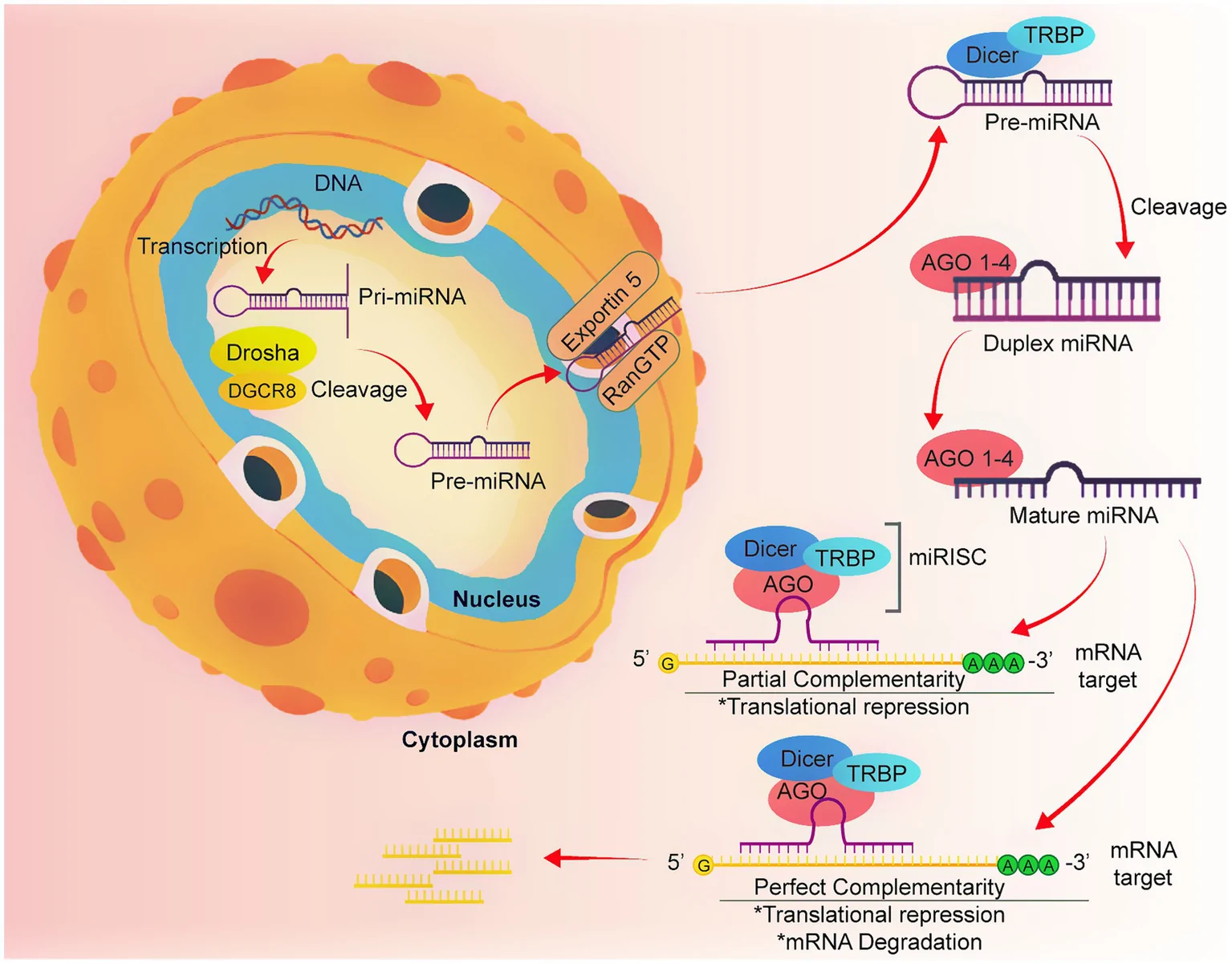
Pathway of miRNA biogenesis in animal cells. In the nucleus, miRNA genes are transcribed into pri-miRNA by RNA polymerase II and cleaved by the microprocessor complex Drosha-DGCR8. The resulting pre-miRNA is then transported to the cytoplasm by exportin-5 and RanGTP. In the cytoplasm, pre-miRNAs are further cleaved into mature miRNA by the enzyme Dicer in complex with TRBP. Functional strands of mature miRNAs are assembled with AGO proteins, while passenger strands are degraded. After strand separation, the miRNA RISC complex facilitates base-pairing interaction between miRNA and mRNA, which leads to suppression of translation or mRNA degradation. DGRC8, DiGeorge syndrome critical region gene 8; pre-miRNA, precursor miRNA; RanGTP, RAS-related nuclear protein-guanosine triphosphate (GTP); AGO, argonaute proteins; RISC, RNA induced silencing complex; TRBP, trans-activation responsive RNA-binding Protein.

Despite the recent advances, our comprehensive understanding of miRNA regulation in bacterial pathogenesis, including their influences on mitophagic pathway activation is still at an early stage. Notably, a large proportion of miRNA-mitophagy regulatory data are obtained from noninfectious disease models, and these findings cannot be directly extrapolated to bacterial infection without empirical experimental validation. The validated miRNA–mitophagy crosstalk during bacterial infection remains limited to a small number of pathogens, and evidence from high-burden pathogens such as *Staphylococcus aureus*, *Escherichia coli*, *Salmonella* spp., and *Helicobacter pylori* remains fragmentary. Therefore, this review mainly contains three-part structure: (1) the roles of miRNAs in bacterial infection, (2) the roles of mitophagy in bacterial infection, and (3) miRNA–mitophagy interplay in infection. Importantly, the regulatory circuit is bidirectional. In addition to host miRNAs controlling host mitophagy, bacterial pathogens deploy T3SS/T4SS-secreted effector proteins, toxins, and other virulence determinants to remodel host mitochondrial homeostasis, mitophagy molecular machinery, and host miRNA expression profiles to promote intracellular bacterial persistence. Moreover, evidence of crosstalk between mitophagic processes and specific miRNAs will provide fundamental insights into the pathophysiology of host-pathogen interactions, thus, we further discuss the potential applications of miRNAs and mitophagy as therapeutic targets for bacterial infection. Given that the versatile functions of miRNAs and mitophagy in the development of infectious disease, it will be fascinating to unveil the mysterious crosstalk in the future.

## 2 The role of microRNA in bacterial infection

Before delving into the complex crosstalk between miRNAs and mitophagy, it is first necessary to outline their individual roles in bacterial infections. Bacterial pathogens are considered to have complicate interplay with pertinent hosts, and the host-pathogen crosstalk is becoming one of the most advanced research of infectious diseases (Zhou et al., 2018). In the context of bacterial infection, miRNAs are probably to regulate host immune pathways that control the magnitude of inflammatory responses via altering various signal pathways (Groeger et al., 2026; Mourenza et al., 2022). In this section, we highlight the latest studies that miRNAs expression is intensely impacted by miscellaneous bacterial pathogens (including *Helicobacter pylori*, *Listeria monocytogenes*, *Mycobacterium tuberculosis*, *Pseudomonas aeruginosa*, *Salmonella*, *Staphylococcus aureus*, and *Escherichia coli* et al.) and are dedicated to the role of miRNAs in regulating cellular activities of the infected cell. These pathogens are epidemiological predominancy in the clinical settings because of their high morbidity, high mortality, and are challenging with limited treatment options or efficient diagnostic tools (Kimura et al., 2023). In addition, these bacterial pathogens not only occupy diverse intracellular and extracellular niches but also employ distinct strategies to manipulate host miRNA expression, thereby providing a comprehensive framework for elucidating the regulatory miRNA networks during infection. While abundant reports describe infection-triggered miRNA dysregulation for most of these pathogens, direct experimental evidence linking these altered miRNAs to mitophagy modulation remains limited for certain species (e.g., *Salmonella* spp., *H. pylori*, *S. aureus*, and *E. coli*). Therefore, we focused on these bacterial pathogens and summarized the current knowledge about the miRNAs involved in bacterial infections in the following section.

### 2.1 Helicobacter pylori

As a usual lifelong and common pathogen, *H. pylori* is able to colonize in human stomach and causes a variety of gastric diseases, including gastric cancer, chronic gastritis, and peptic ulcers worldwide (Crowe, 2019; Li et al., 2025). It is now well-established from numerous studies, that the expression profiles of miRNAs were altered in gastric epithelial cells in the process of *H. pylori* infection, including miR-1289, miR-146a, miR-152/miR-200b, miR-155, miR-16, miR-210, miR-221, miR-375 and lethal-7 (let-7) family (Kalani et al., 2021; Ramesh et al., 2023; Ranjbar et al., 2018; Săsăran et al., 2021; Xie et al., 2017). As the first investigated miRNA in human, let-7 and its family consist of more than 10 members. The let-7b levels were decreased by *H. pylori* infection, and subsequently upregulating the expression of TLR4 and NF-κB, finally inducing the host inflammation (Teng et al., 2013). In regard to the miRNAs induced by *H. pylori*, miR-155 was one of the most well-recognized miRNAs, and the higher levels of miR-155 were detected in gastric tissue of patients, who is infected with *H. pylori* (Blosse et al., 2019). On the one hand, the expression of miR-155 is upregulated by secretion of tumor necrosis factor-α (TNF-α) in responses to bacterial infections. On the other hand, miR-155 is participated in a negative feedback response via reducing NF-κB expression and attenuating the production of pro-inflammatory cytokines during *H. pylori* infection. In addition, miR-155 is remarkably expressed in activated B and T cells presented in conditions of chronic gastric inflammation (Săsăran et al., 2021). Furthermore, several other miRNAs including miR-204, miR-30b, and miR-27b were abnormally expressed in *H. pylori*-infected cells and altering neoplastic transformation (Du et al., 2020; Geng et al., 2016; Zhou and Xu, 2019). Altogether, these reports demonstrated the roles of miRNAs in modulating pathogenesis of diverse host cell types infected by *H. pylori*.

### 2.2 Listeria monocytogenes

*L. monocytogenes* is a well-known intracellular Gram-positive and ubiquitous foodborne bacterium that causes a systemic disease listeriosis which is resulting from the ingestion of polluted food (Huang et al., 2026; Tang et al., 2023). This flexible pathogen spreads from the gastrointestinal tract to cross intestinal, blood–brain, and placental barriers and evades miRNA-mediated defense in host cells (Drolia and Bhunia, 2019; Du et al., 2018). Up to now, accumulative studies have established that several miRNAs such as miR-146b, miR-155, miR-16, miR-145, and let-7a1 were dysregulated under *L. monocytogenes* infection in different cell types (Allahverdy and Rashidi, 2023; Cassidy et al., 2020; Sesto et al., 2014; Zhang et al., 2018). For instance, a recent study showed that miR-155 is required for development of CD8^+^ Tissue-resident memory T cells (T_RM_) response during systemic *L. monocytogenes* infection (Cassidy et al., 2020). In another major study, infection of mice with *L. monocytogenes* downregulated miR-29 expression and impaired the immune responses of CD4+ T cells and CD8+ T cells via targeting interferon-γ (Ma et al., 2011). Interestingly, a follow-up investigation found that miR-21 regulated phagocytosis to control *L. monocytogenes* infection via weakening the intracellular niche (Johnston et al., 2017). The intestinal tract is considered as a main pool of microbes in humans, but how the intestinal microbiota modulated miRNA induction remains largely unknown. The preliminary evidence demonstrated that intestinal microbiota altered the expression of miR-378, miR-143, miR-148a, miR-200b, and miR-200c in mice gut after orally Listeria infection with *L. monocytogenes* (Archambaud et al., 2013). Besides, other study also reported similar findings that miRNAs provided the immune protection by regulated cellular processes such as proliferation, tight junction proteins, and inflammation in response to bacterial infection (Morris and Choudhry, 2021; Yee Mon et al., 2024). Taken together, these study present new evidences for understanding the role of miRNAs in host–microbe interaction induced by *L. monocytogenes* infection.

### 2.3 Mycobacterium

Tuberculosis (TB) is a rigorous infectious disease and is a leading cause of death worldwide from a single infectious pathogen before the present coronavirus disease (COVID-19) pandemic (Diao et al., 2023; Zhu et al., 2021). As the main pathogen of TB, *Mycobacterium tuberculosis* (*M. tuberculosis*) is a predominant human pathogen that has coevolved with hosts for more than thousands of years (Gopalaswamy and Subbian, 2026). Except for *M. tuberculosis*, other intracellular bacteria *Mycobacterium bovis* (*M. bovis*) also infects animals and humans and is one of the well-recognized mycobacteria (Lange et al., 2022). It has been conclusively shown that miRNAs are the key regulators of various biological processes during *M. tuberculosis* infection, such as autophagy, apoptosis, and inflammatory response (Fu et al., 2020; Hackett et al., 2020; Liang et al., 2018; Wu et al., 2024). For example, the expression of miR-18b was downregulated by *M. tuberculosis* infection, which subsequently increased the expression of target gene hypoxia-inducible factor 1α (HIF-1α) and triggered the signaling pathways of p38 MAPK to enhance secretion of several inflammatory cytokines and to decrease bacterial burdens in macrophages (Zhu et al., 2021). Regarding inflammatory responses, a relative restudy indicated that *M. tuberculosis* infection could alter the host immunometabolic programming by miR-21 to evade inflammation and ensure survival in macrophage (Hackett et al., 2020). As a chief miRNA for immune responses, miR-155 is also overexpressed in macrophages in the process of *M. tuberculosis* and *M. bovis* infection. Enforced the expression of miR-155 enhances autophagic influx, thus accelerating mycobacterial phagosome maturation and declining the viability of intracellular bacteria in macrophage (Rothchild et al., 2016). In addition, infection of macrophages with *M. tuberculosis* induced higher expression of miR-125a, miR-132, miR-146a, miR-206, miR-26a, and miR-let-7, which functioned as important regulators involved in TB pathogenesis and modulated host innate and adaptive immunity (Sinigaglia et al., 2020). Consequently, miRNAs are probably potent mediators of inflammatory responses in macrophage with regard to mycobacteria invasions and provide the promising therapeutic targets for controlling TB.

### 2.4 Pseudomonas aeruginosa

As a versatile and opportunistic Gram-negative bacterial pathogen, *P. aeruginosa* is able to cause both acute and chronic infections in a broad range of individuals, especially those with compromised immune systems such as cystic fibrosis (CF) (Feng et al., 2026; Zhao et al., 2019; Zhao, K. et al., 2023). *P. aeruginosa* also contributes to the pathogenesis of chronic obstructive pulmonary disease (COPD), which severely affects almost 10% of the world’s population and is considered as the third leading cause of death around the world (Huang et al., 2022; Song et al., 2021). Up to now, a large number of studies have identified the important role of miRNAs in modulating inflammatory responses and host defense during *P. aeruginosa* infections (Fang et al., 2024; Gabaldón et al., 2020; Koeppen et al., 2021; McClellan et al., 2021; Sarkar et al., 2025; Shi et al., 2021). The cooperated laboratory previously demonstrated that miR-302b was induced by ERK-p38-NF-κB pathways under *P. aeruginosa* infection (Zhou et al., 2014). The miR-302b is a vital regulator of TLR-induced NF-κB signaling including macrophage activation and respiratory inflammation via targeting of IL-1R-associated kinase 4 (IRAK4) for NF-κB activation. In addition, the latest research indicated that enhanced overexpression of miR-302/367 cluster promoted mitophagic response and reduced production of ROS (Huang et al., 2020b). Notably, miR-302/367 clusters regulate host mitophagy by targeting NF-κB in the process of *P. aeruginosa* invasion, thereby accelerate the elimination of intracellular bacterium and maintain cell homeostasis. Furthermore, our cooperated laboratory identified another important miRNA (miR-301b), which modulated the production of the anti-inflammatory cytokines IL-4 and TGF-β1 by repressing c-Myb expression against *P. aeruginosa* infection (Li et al., 2016). As a predominant miRNA for immune responses, let-7 also altered host innate response for combating *P. aeruginosa* PA14 infection in a *P. aeruginosa*-infected *Caenorhabditis elegans* model (Zhi et al., 2017). The let-7 functions as a critical regulator in innate immune response pathways to induce stronger immunity and diminishing pathogen-induced stress during *P. aeruginosa* infection (Ren and Ambros, 2015). Interestingly, let-7b, an important member of let-7 family, is able to target genes in *P. aeruginosa* to alter gene expression, thereby reduce the formation of biofilm formation and increase beta-lactam sensitivity (Koeppen et al., 2021). There are several other miRNAs, such as hsa-miRNA-432-5p, hsa-miRNA-449c, hsa-miR-548, hsa-miRNA-1247, hsa-miRNA-1276, and hsa-miRNA-3170, whose levels are differentially expressed in CF patients infected with *P. aeruginosa* (Fesen et al., 2019). With regard to the crucial role of miRNAs when it comes to response the *P. aeruginosa* infection, future more mechanism studies will determine the potentially therapeutic efficacies of well-recognized miRNAs in the treatment of *P. aeruginosa-*related diseases.

### 2.5 Salmonella

*Salmonella* is a group of bacteria that frequently cause food-related illness called salmonellosis in animals and humans, posing a severe threat to public health worldwide (Shen et al., 2021). All Salmonella belong to the family Enterobacteriaceae, which consists of three main members including *S. typhi*, *S. typhi*murium, and *S. enteritidis*, and thrive in the intracellular niche for their successful propagation in host cells (Knodler and Elfenbein, 2019). It has been established that the dysregulation of several miRNAs was relevant the pathogenesis of *Salmonella*-induced disease (Aguilar et al., 2021; Aguilar et al., 2020; Huang et al., 2019; Huang et al., 2023; Wu et al., 2017; Xu et al., 2021). It is well known that cells infected with bacteria contribute to eliminate infections via activate specific signals that instruct contiguous cells to amplify the pro-inflammatory cytokine response. In a latest study, the researchers demonstrated that the endoplasmic reticulum stress response is induced in both *Salmonella*-infected and bystander cells, and this initiates the activation of JNK signal pathway, repression of transcription factor E2F1 (E2 promoter binding factor 1), and consequent reprogramming of miRNA expression such as miR-15a-5p, miR-16-5p, miR-421, miR-744-5p, and let-7i-3p (Aguilar et al., 2021). Notably, they further found that downregulated expression of E2F1 and miRNAs alterations promoted *Salmonella* replication in the infected cells and bystander cells. A recent literature showed that miR-139-5p was able to decline the expression of IL-1β and TNF-α to inhibit host inflammatory responses and oxidative stress by targeting TNF receptor associated factor 6 (TRAF6) in *Salmonella*-infected macrophages (Xu et al., 2021). Except for the great threat to human health, *Salmonella* is also able to induce miRNAs production and regulate the innate immunity in animals. A comparative transcriptomic analysis of upregulated mRNAs in peripheral blood indicated that miR-146a was likely to participate in regulation of progression of *S. typhimurium* infection in pigs (Huang et al., 2019). Additionally, miRNAs including gga-miR-34a-5p, gga-miR-125b-5p, gga-miR-1416-5p, and gga-miR-1662 may manipulate the innate immunity in the course of *S. enteritidis* infection in laying hens (Wu et al., 2017). Hence, these upregulated miRNAs may provide a novel strategy for controlling *Salmonella*-induced inflammation. To date, multiple studies have reported changes in miRNA expression during *Salmonella* infection, and mitophagy has been confirmed to restrict or support *Salmonella* intracellular survival in host cells. Nevertheless, direct experimental evidence connecting dysregulated host miRNAs to mitophagy modulation during *Salmonella* infection is still lacking; most reported miRNA functions are restricted to inflammatory response regulation without assessment of mitophagy-related outputs.

### 2.6 Other bacterial pathogens

In addition to the bacterial infection models as mentioned above (Figure 2), miRNAs have been contributed to regulate host immune responses for fighting the other well-recognized bacteria. As one of the most important pathogens, *Staphylococcus aureus* (*S. aureus*) is found in the nasal mucosa of more than 20% of the general population worldwide and occasionally causes endocarditis, pneumonia, and skin infections (Lee et al., 2018). Several miRNAs such as miR-128, miR-223, miR-142, and miR-155 have been investigated to play an important role in pathogenesis of *S. aureus* infections in both humans and animal models (de Kerckhove et al., 2018; Ma et al., 2019; Mirzaei et al., 2020; Tanaka et al., 2017). As a case in point, skin wound healing was impaired in the mice with deficient miR-142 following *S. aureus* infection, and the expression of the GTPases were remarkably enhanced in miR-142-deficient neutrophils (Tanaka et al., 2017). By far, *S. aureus* is the most clinically relevant in humans, while it is also able to induce miRNAs production and regulate the innate immunity in large animals. A latest study found that miR-1301 and miR-2284r was significantly regulated in peripheral blood of dairy cows after *S. aureus* infection. These important miRNAs were predicted to involve in several crucial immune system-related pathways such as chemokine signaling pathway (Luoreng et al., 2021). miR-15a/16 have been identified to function as a key regulator in bacterial induced-sepsis. Deletion of miR-15a/16 altered phagocytosis and *Escherichia coli* elimination by targeting TLR40-related pathways in sepsis mice (Moon et al., 2014). In *Porphyromonas gingivalis* (*P. gingivalis*)-infected bone marrow-derived macrophages, mmu-miR-155-5p markedly declined the secretion of TNF-α. Likewise, injection of mmu-miR-155-5p reduced lesion size in a *P. gingivalis*-infected mouse model (Huck et al., 2017). Filopodia, the thin actin-rich cell protrusions are essential for phagocytosis and bacterial internalization by forming host-cell filopodia. It is reported that miR-29b-2-5p strongly increased filopodia in hosts via inhibiting its target gene UNC5C during *Shigella flexneri* infection (Sunkavalli et al., 2017). miR-141-3p expression was highly reduced in *Streptococcus pneumoniae*-infected rats and astrocytes. Overexpression of miR-141-3p could decline the production of several important inflammatory cytokines including TNF-α, IL-1β, and IL-6 by directly targeted HMGB1 (Fang et al., 2021). These studies pinpoint the multifaceted transcriptional and posttranscriptional mechanisms of cells via regulation miRNAs response to bacterial infection. *S. aureus* sepsis and *E. coli*-caused urinary/abdominal infections are well documented to engage host mitophagy for host defense. Although multiple miRNAs are dysregulated upon *S. aureus* and *E. coli* infection, very few studies have functionally linked these miRNAs to mitophagy machinery. Further experimental work is required to dissect the miRNA-mitophagy axes during *S. aureus* and *E. coli* infections.

**Figure 2:**
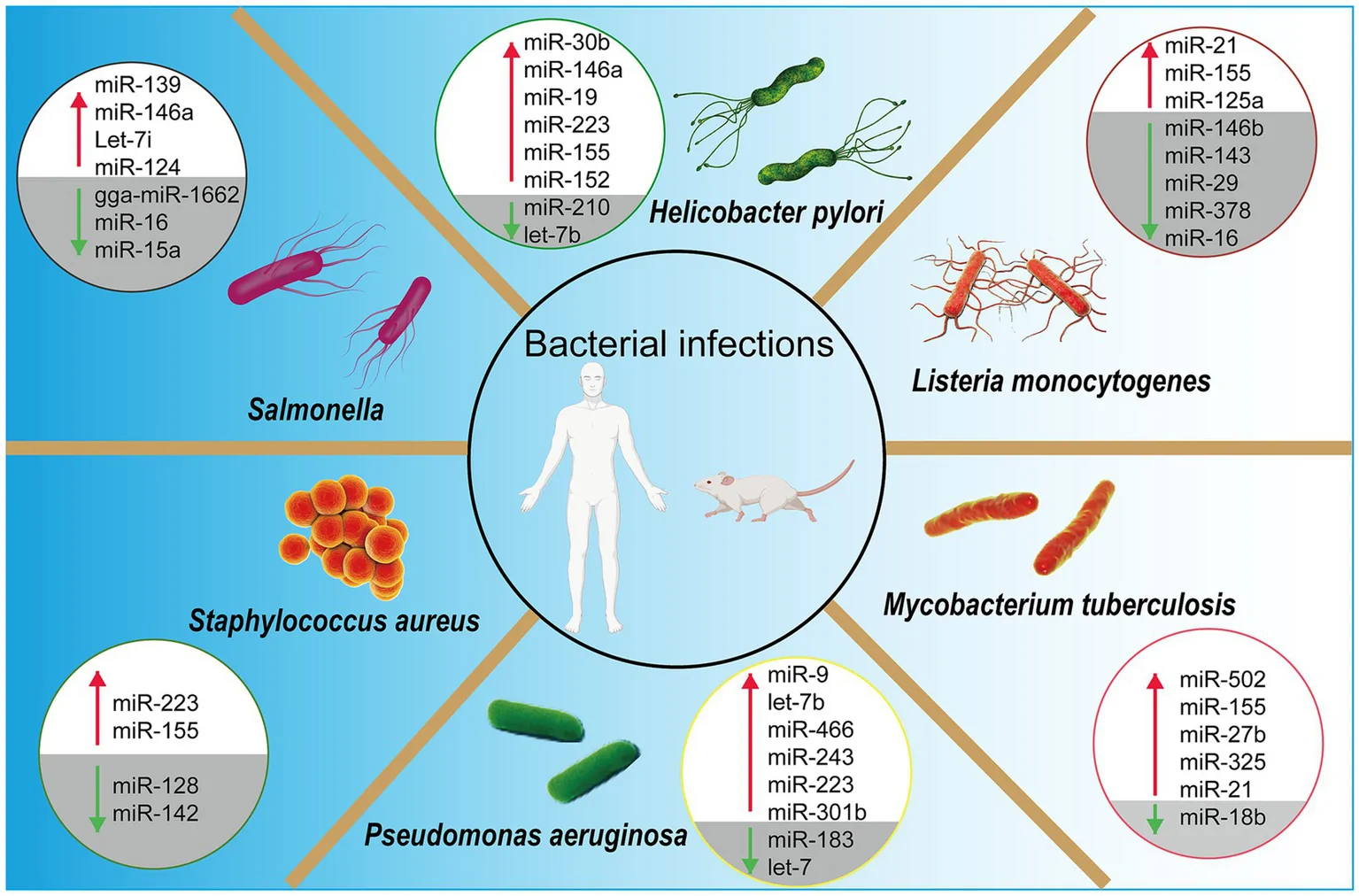
MiRNA modulation upon infection by the different bacterial pathogens in humans and mice. Summary of the expression profile of miRNAs for 6 different bacteria in human and mice, as discussed in the main text. The up-regulated miRNAs are indicated by red arrows and the down-regulated miRNAs are presented by green arrows.

Despite extensive descriptive data, most miRNA studies in bacterial infections rely on transformed cell lines or murine models that do not accurately reflect human pathophysiology. Functional causality is often inferred from target prediction rather than demonstrated through genetic perturbation, and contradictory findings for the same miRNA across different pathogens (e.g., miR-155) remain largely unexplained. Methodological heterogeneity in bacterial strains, infection times, and normalization strategies further impedes cross-study comparisons. Rigorous validation in primary human cells and standardized experimental frameworks are urgently needed to distinguish functionally relevant miRNAs from mere inflammatory byproducts.

The studies discussed above highlight the diverse transcriptional and post-transcriptional mechanisms by which cells employ miRNAs to combat bacterial infection. Because many of these miRNAs regulate pathways critical for cellular homeostasis, they likely also intersect with mitophagy, a central mitochondrial quality-control process. Therefore, we next turn to an overview of mitophagy and its role in host defense against bacterial pathogens.

## 3 Mitophagy pathway and its role in bacterial infection

### 3.1 PINK1/Parkin-mediated mitophagy

As the most classical pathway of mitophagy, the phosphatase and tensin homologue (PTEN)-induced putative kinase 1 (PINK1)-Parkin mediated mitophagy is widely investigated, which is recruited to elimination of defective or damaged mitochondria (Palikaras et al., 2018). PINK1 is a serine/threonine kinase and can be delivered to the inner mitochondrial membrane (IMM) under a normal cellular activity. After cleaved by the specific mitochondrial-related peptidase and presenilin-associated rhomboid-like protease, the PINK1 protein is subsequently degraded via a ubiquitin-mediated proteasome system (Sekine and Youle, 2018). However, the translocation of PINK1 into the IMM is blocked under challenged conditions, accelerating the stabilization of PINK1 on the outer mitochondrial membrane (OMM) of the impaired mitochondria (Song et al., 2020). Afterwards, PINK1 recruits the E3-ubiquitin ligase Parkin to the mitochondrial surface and enhances the E3 ligase activity of Parkin by phosphorylating Parkin and ubiquitin (Harper et al., 2018). The damaged mitochondria are labeled with Parkin, which promoting ubiquitination of several important OMM proteins, including voltage-dependent anion channel-1 (VDAC1), mitofusin 1 and 2 (MFN1/2), Miro 1, or TOM20 (Liu et al., 2023; Palikaras and Tavernarakis, 2012; Sun et al., 2021). Ub-tagged OMM proteins attach to ubiquitin-binding domain of autophagy receptors, such as p62, neighbor of BRCA1 gene (NBR1), Nuclear Dot Protein 52 (NDP52), TAX1 binding protein 1 (TAX1BP1), and optineurin (OPTN), which collaborate with LC3-interacting region (LIR) of LC3 to initiate mitophagy (Heo et al., 2015). Reliable experimental methods can be used to verify mitophagy rather than general autophagy, including: (i) selective loss of mitochondrial proteins (e.g., TOM20, TIM23, VDAC1) in the presence of lysosomal inhibitors; (ii) colocalization of mitochondrial markers with autophagosomal/autolysosomal markers (e.g., LC3/LAMP1); (iii) mitophagy flux measurements using mitochondrial reporters (e.g., mt-Keima, mito-QC); and (iv) assessment of mitochondrial depolarization (e.g., JC-1, TMRM) followed by lysosomal degradation (Eldeeb et al., 2024; Sun et al., 2017). The accumulation of LC3-II alone is not sufficient evidence of mitochondrial autophagy, as it reflects the activation of general autophagy.

### 3.2 Receptor-mediated mitophagy

Mitophagy receptors are mainly located on the OMM of cells, which directly interact with the LIR motifs of LC3 and engulf the damaged mitochondria to activate mitophagy (Gatica et al., 2018). The first mitophagy receptor, autophagic related protein 32 (Atg32) was found in *Saccharomyces cerevisiae* for mitochondrial turnover (Gatica et al., 2018). In the following years, many mitophagy receptors on the OMM have been determined, including NIX (NIP3-like protein X), BNIP3 (BCL2 interacting protein 3), FUNDC1 (FUN14 domain-containing protein 1), and NLRX1 (NOD-like receptor X1) (Bi et al., 2024; Palikaras et al., 2018). Notably, cardiolipin and prohibitin 2 (PHB2) have been determined as the novel mitophagy receptor in the IMM, mediating mitochondrial removal upon different stress conditions (Shen et al., 2017; Wei et al., 2017). These mitophagy receptors play a central role in regulating dysfunctional mitochondria and preserving cellular homeostasis. Nevertheless, the detail mechanism of IMM mitophagy receptors in maintaining mitochondrial function remains largely unknown (Yan et al., 2020), and further studies is required to gain a deeper understanding of the key function of IMM receptors in mitophagy.

### 3.3 The interplay between mitophagy and bacterial infection

Given the key role of mitophagy in regulating various cellular activities, their specific role in manipulating host response to the bacterial infection is conceivable. A great number of studies have investigated the disruption of mitophagic functions upon bacterial infections (Mahla et al., 2021; Mirza et al., 2023; Nan et al., 2024; Sun et al., 2025). Below we discuss how different pathogens target the mitophagy to impact their intracellular survival and dissemination by mediating host immunity. Beyond host-originated signaling, bacterial pathogens deploy various secreted toxins and effector proteins (translocated via T3SS, T4SS, or other secretion apparatuses) to actively remodel host mitochondrial integrity, mitophagy-related protein function, and even the transcription and processing of host miRNAs. Such bacterial-driven modifications can either trigger or suppress mitophagy, ultimately favoring bacterial replication and persistence inside host cells. Importantly, mitophagy has dual, context-dependent outcomes during bacterial infections. On one hand, physiological mitophagy clears damaged mitochondria, curtails ROS/mtDNA release, and restrains overactivated innate immune responses to protect host tissues. However, excessive mitophagy induces massive mitochondrial depletion and host cell death, aggravating tissue injury during infection. In addition, multiple intracellular bacterial pathogens exploit mitophagy to dampen antimicrobial responses and promote their own intracellular survival (Li, J. et al., 2026). We illustrate this duality using three representative pathogens: *P. aeruginosa*, *M. tuberculosis*, and *L. monocytogenes*.

#### 3.3.1 *P. aeruginosa* infections

In the past decade, a number of researchers have made great efforts to determine the balance between mitophagy and host defense during bacterial infection. For instance, *P. aeruginosa* infection caused mitochondrial damage by a type III secretion apparatus (T3SS), leading to production of ROS and release of mtDNA, activating NLRC4 inflammasome activation (Jabir et al., 2015). In the meantime, host mitophagy was triggered to eliminate damaged mitochondria and downregulated NLRC4 inflammasome activation (Jabir et al., 2015). Several other studies have shown the protective function of mitophagy during *P. aeruginosa* infection via diminishing the innate immune response (Kang et al., 2018; Kirienko et al., 2015). As an important iron-binding siderophore toxin of *P. aeruginosa*, pyoverdin regulated mitochondrial homeostasis to initiate mitophagy in *C. elegans.* Consequently, mitophagy activation provided protection against *P. aeruginosa* by treated with phenanthroline (Kirienko et al., 2015). In addition, pyoverdin caused host mitochondrial damages and disrupting mitochondrial function and triggering mitophagy, while the *C. elegans* initiated a specific host defense against *P. aeruginosa* infection by mediated the ethanol and stress response network (Kang et al., 2018). In one of our published studies, we found that the miR-302/367 cluster up-regulated upon *P. aeruginosa* infection induces the PHB2-dependent mitophagy pathway to enhance bacterial clearance via a negative regulation of NF-kB (Huang et al., 2020b). Recent evidence suggests that cigarette smoking dampened *P. aeruginosa*-induced mitophagy flux, aggravated host mitochondrial injury and NLRP3 inflammasome activation in alveolar macrophages (White et al., 2022). Afterwards, inhibiting expression of caspase-1 reduced CS primed *P. aeruginosa*-triggered acute pulmonary injury and increased mice survival (White et al., 2022). Notably, a recent discovery pinpoint that cyclic GMP-AMP synthase is vital for *P. aeruginosa*-induced mitophagy by detecting the mtDNA and regulating inflammasome activation and TLR9 signaling (Wang et al., 2022). However, the role of mitophagy in regulation of inflammasome activation upon infection with other bacteria has not been wildly addressed. Moreover, quorum sensing of *P. aeruginosa* also play a key role in regulating mitochondrial dysfunction and mitophagy pathway of host upon infection (Aggarwal et al., 2024; Mirza et al., 2023). Notably, bacterial virulence determinants, including T3SS secretion system effector proteins and pyoverdin siderophores, trigger mitochondrial injury and initiate host responses. These bacterial products act as upstream drivers, perturbing mitochondrial homeostasis before mitophagy activation, which might be potentially beneficial to the pathogen for bacterial viability.

#### 3.3.2 *Mycobacterial* infections

Mitophagy pathway has been implicated in host defense against intracellular pathogens such as *M. tuberculosis* (Das et al., 2026). For example, disruption of the mitophagy pathway by knockout of *parkin* genes increased *M. tuberculosis* replication, while overexpression of Parkin in macrophages significantly decreased bacterial burden (Manzanillo et al., 2013). In an early study, a novel mediator of mitophagy Smurf1 was identified by a genome-wide siRNA screen, which played a key role in the Parkin-mediated mitophagy (Orvedahl et al., 2011). This finding was confirmed in follow-up study. Smurf1 is an important bridging molecule to recruit NBR1 and LC3, and mediates ubiquitin-dependent selective autophagy against *M. tuberculosis* infection *in vitro* and *in vivo* (Franco et al., 2017). Several recent discoveries in relation to the regulation of mitophagy have suggested potential therapeutic strategies to limit *M. tuberculosis* growth or infection (Bird et al., 2020; Lee et al., 2020; Mahla et al., 2021). For example, imiquimod could control intracellular *M. tuberculosis* growth by increasing mitochondrial ROS and promoting BNIP3-mediated mitophagy in macrophages (Lee et al., 2020). Thiopeptide antibiotics are emerging clinical compounds that induced mitophagy in an infected macrophages model and exhibited potent activity against intracellular *M. tuberculosis* (Bird et al., 2020). In addition, *M. tuberculosis* infection strongly induced NIX-mediated mitophagy, which initiated host metabolic reprogramming and promoted differentiation of macrophage to M1 phenotype, indicating the key role of mitophagy in understanding anti-tuberculosis immune mechanism (Mahla et al., 2021). Notably, an interesting study pinpointed that *M. tuberculosis* was able to directly cause mitochondrial injury in infected macrophages and influence the expression of mitophagy-related genes to manipulate host susceptibility to mycobacterial infection (Patrick and Watson, 2021). Because *M. tuberculosis* can secrete multiple effector proteins to induce mitochondrial damage and modulate mitophagy signaling, which constitutes a critical strategy for mycobacterial intracellular persistence. Recent reviews have emphasized that BNIP3/NIX-mediated mitophagy serves as a promising therapeutic target for tuberculosis immunotherapy, as its activation enhances macrophage bactericidal activity and limits intracellular mycobacterial survival (Gao et al., 2025). Future studies should focus on how mitophagy-triggered innate immune responses influence the progress of tuberculosis disease (Lee et al., 2023; Song et al., 2022), which might develop host-directed therapeutics and promote positive feedback from tuberculosis patient.

#### 3.3.3 *Listeria* infections

Under infection condition, mitophagy receptors orchestrate host defense by targeting the critical innate immune signaling factors (Mitchell et al., 2018). An attractive example is the intracellular bacterium, called *L. monocytogenes*. Infection with *L. monocytogenes* enables mitochondrial fission and causes fragmentation of the mitochondrial network by the pore-forming toxin listeriolysin O (Stavru et al., 2011). Knocking down the key components of the fission (Drp1) and fusion (Mfn1 and Mfn2) system alters the survival of *L. monocytogenes* inside the host cells (Stavru et al., 2011). Notably, a recent study found that this process is also associated with the Mic10 protein, which is essential for *L. monocytogenes*-induced mitochondrial fragmentation and early infection (Carvalho et al., 2020). In a late infection stage, *L. monocytogenes* is able to induce mitophagy in macrophage by listerolysin O. This important process is mediated by a new mitophagic receptor, NLRX1, which is indispensable for *L. monocytogenes*-induced mitophagy (Zhang et al., 2019). Interestingly, NLRX1 deficiency increased production of mitochondrial ROS (mtROS) and bacterial clearance, thereby promoted the survival rate of mouse (Zhang et al., 2019). The pore-forming virulence factor listeriolysin O (LLO) produced by *L. monocytogenes* drives mitochondrial fragmentation and subsequent NLRX1-dependent mitophagy, demonstrated that how bacterial toxin reprogram host mitophagy and how mitophagy can inadvertently enhance bacterial viability. Therefore, targeting mitophagy machinery will provide an efficient therapeutic strategy against *L. monocytogenes* infections.

The functional outcome of mitophagy during infection is pathogen-specific, conferring protection against *M. tuberculosis* and *P. aeruginosa*. The interplay between PINK1/Parkin and receptor-mediated pathways remains poorly characterized, and their relative contributions across cell types and infection stages are largely unexplored. Most studies have relied on acute models and single-time-point measurements, overlooking chronic infection dynamics. Accordingly, real-time reporters and cell-type-specific knockouts are needed to define indispensable functions and circumvent pharmacological artifacts. Although the studies discussed above confirm the importance of mitophagy in host defense, the upstream regulatory mechanisms that govern this process, particularly during bacterial infection, remain poorly understood. The following section therefore explores how miRNAs serve as critical modulators of the mitophagy pathway in the context of infection or other pathophysiological settings.

## 4 Mitophagy regulation by microRNA

As mentioned previously, the mitophagy process is mediated by some central proteins of the OMM or the IMM following infection with bacterial pathogens and miRNAs could regulate host-pathogen interplay in response to bacterial infections (Mourenza et al., 2022). Although a number of studies highlighted the miRNAs associated with the regulation of autophagic pathways (Peng et al., 2025; Silwal et al., 2020; Zhang et al., 2023), the roles of miRNAs in the regulation of mitophagy pathway upon bacterial infection has not been extensively investigated. Therefore, here we focus on recent advances with regard to the interplay between miRNAs and mitophagy in several contexts.

### 4.1 Interconnecting miRNAs and PINK1/Parkin-mediated mitophagy

An earlier study has determined the key role for miR-27a and miR-27b in the regulation of mitophagy (Kim et al., 2016). Particularly, miR-27a and miR-27b were shown to suppress PINK1-medieated mitophagy by reduced ubiquitin phosphorylation and LC3-II accumulation in the fragmental mitochondria (Kim et al., 2016). In the same year, an interesting study found that miR-181a overexpression also inhibited mitophagy by binding Parkin and impeded the colocalization of mitochondria and autophagosomes. Attenuated the expression of miR-181a was able to accelerate mitophagy process in neuroblastoma cells (Cheng et al., 2016). In a recent published article, miR-218 was recognized as another microRNA targeting Parkin, and negatively regulated PINK1/PRKN-mediated mitophagy. Overexpression of miR-218 decreased the mRNA level of Parkin, which subsequently deregulated the E3 ubiquitin ligase activity and mitochondria co-localization with LC3-II, thus inhibited mitochondrial clearance (Di Rita et al., 2020). Besides, miR-140-5p was also shown to suppress Parkin expression and altered mitochondrial translocation, thereby blocked mitophagy process via the HIF-1α/miR-140-5p/Parkin axis in a kidney injury model (Zhang, Q. et al., 2022). Since dysfunctional mitophagy are associated with diverse human disorders, these discoveries reveal a key link between microRNA and PINK1/Parkin-mediated mitophagy, which provides a potential therapeutic approach for mitochondrial-related diseases (Zhou et al., 2023).

### 4.2 Interconnecting miRNAs and receptor-mediated mitophagy

In the past few years, several studies revealed a link between miRNAs and receptor-mediated mitophagy (Chevalier et al., 2022; Dabin et al., 2022; Kim et al., 2021; Li et al., 2018; Li et al., 2014; Liu et al., 2019). For instance, miR-137 was identified as a hypoxia-responsive microRNA, which significantly inhibited mitophagy process (Li et al., 2018; Li et al., 2014). In detail, miR-137 suppressed mitophagy in a hypoxia-induced condition via targeting two important mitophagy receptors FUNDC1 and NIX. Such effect could be rescued by transfecting FUNDC1 and NIX expression vectors due to a lack of the miR-137 binding sites (Li et al., 2014). In the following year, an interesting study found that miR-145 was an upstream regulator of mitophagy by binding Bnip3 and causing mitophagy dysfunction. Nevertheless, reduction of miR-145 expression reversed mitophagy activity and remarkably increased SH-SY5Y cell viability during TNFα-mediated inflammation injury (Liu et al., 2019). Similarly, in another major study, Kim et al. found that miR-126-5p functioned as a key regulator of peroxisome proliferator-activated receptor-gamma co-activator 1-alpha (PGC1α). Enforced expression of miR-126-5p altered Bnip3-independent mitophagy and stimulated cartilage degradation (Kim et al., 2021). Amongst the miRNAs as mentioned above (Table 1; Figure 3), the other miRNAs (such as miR-30a-5p, miR-let-7e, miR-24-3p) have been contributed to in regulation of receptor-mediated mitophagy in different contexts including BNIP3L/NIX-dependent mitophagy or PHB2-dependent mitophagy (Chevalier et al., 2022; Dabin et al., 2022; Zhang, Y. et al., 2022).

**Table 1:** List of microRNAs identified during bacterial infection and their mechanisms of action.

Bacteria	miRNA	Up/down-regulation	Targeted cellular function	References
*Helicobacter pylori*	miR-1289	up	gastric acid secretion	Kalani et al. (2021)
	miR-152/miR-200b	down	immune suppression	Xie et al. (2017)
	miR-146a/miR-55	up	apoptosis	Săsăran et al. (2021)
	miR-210	down	cell proliferation	Kiga et al. (2014)
	miR-19/miR-21	up	IRAK1 pathway	Ranjbar et al. (2018)
	miR-21/miR-223	up	NF-kB pathways	Zhang et al. (2014)
	let-7b	down	inflammation	Teng et al. (2013)
	miR-155	up	NF-kB pathways	Blosse et al. (2019)
	miR-204	down	inflammatory processes	Du et al. (2020)
	miR-27b	up	gastric mucosal injury	Geng et al. (2016)
*Listeria monocytogenes*	miR-125a	up	NF-kB pathways	Drolia and Bhunia (2019)
	miR- 146b/miR-16	down	inflammatory responses	Du et al. (2018)
	miR-155	up	T cell responses	Cassidy et al. (2020)
	miR-29	down	immune responses	Ma et al. (2011)
	miR-21	up	phagocytosis	Johnston et al. (2017)
	miR-378/miR-143	down	intestinal homeostasis	Archambaud et al. (2013)
*Mycobacterium tuberculosis*	miR-18b	down	inflammatory responses	Zhu et al. (2021)
	miR-502	up	NF-kB pathways	Liu et al. (2021)
	miR-155	up	autophagy	Rothchild et al. (2016)
	miR-27b	up	apoptosis	Liang et al. (2018)
	miR-325	up	STAT3 pathways	Fu et al. (2020)
	miR-21	up	glycolysis	Hackett et al. (2020)
*Pseudomonas aeruginosa*	miR-9	up	NF-kB pathways	Shin et al. (2017)
	let-7b	up	protein expression	Koeppen et al. (2021)
	miR-183/96/182 cluster	down	Innate immunity	McClellan et al. (2021)
	miR-466	up	phagocytosis	Shi et al. (2021)
	miR-243	up	dauer formation	Gabaldón et al. (2020)
	miR-223	up	inflammation	Fang et al. (2024)
	miR-302b	up	TLR pathways	Zhou et al. (2014)
	miR-302/367 cluster	up	mitophagy	Huang et al. (2020b)
	miR-301b	up	inflammatory response	Li et al. (2016)
*Salmonella*	miR-15a/miR-16	down	JNK pathway	Aguilar et al. (2021)
	miR-139	up	inflammatory response	Xu et al. (2021)
	miR-146a	up	inflammatory response	Huang et al. (2019)
	Let-7i	up	endolysosomal trafficking	Aguilar et al. (2020)
	miR-124	up	MAPK pathway	Huang et al. (2023)
	gga-miR-34a	down	metabolism and immunity	Wu et al. (2017)

IRAK1, interleukin 1 receptor-associated kinases; JNK, c-Jun NH (2)-terminal kinases; MAPK, mitogen-activated protein kinase; NF-kB, nuclear factor kappa-B; STAT3, signal transducer and activator of transcription 3; TLR, toll-like receptor.

**Figure 3:**
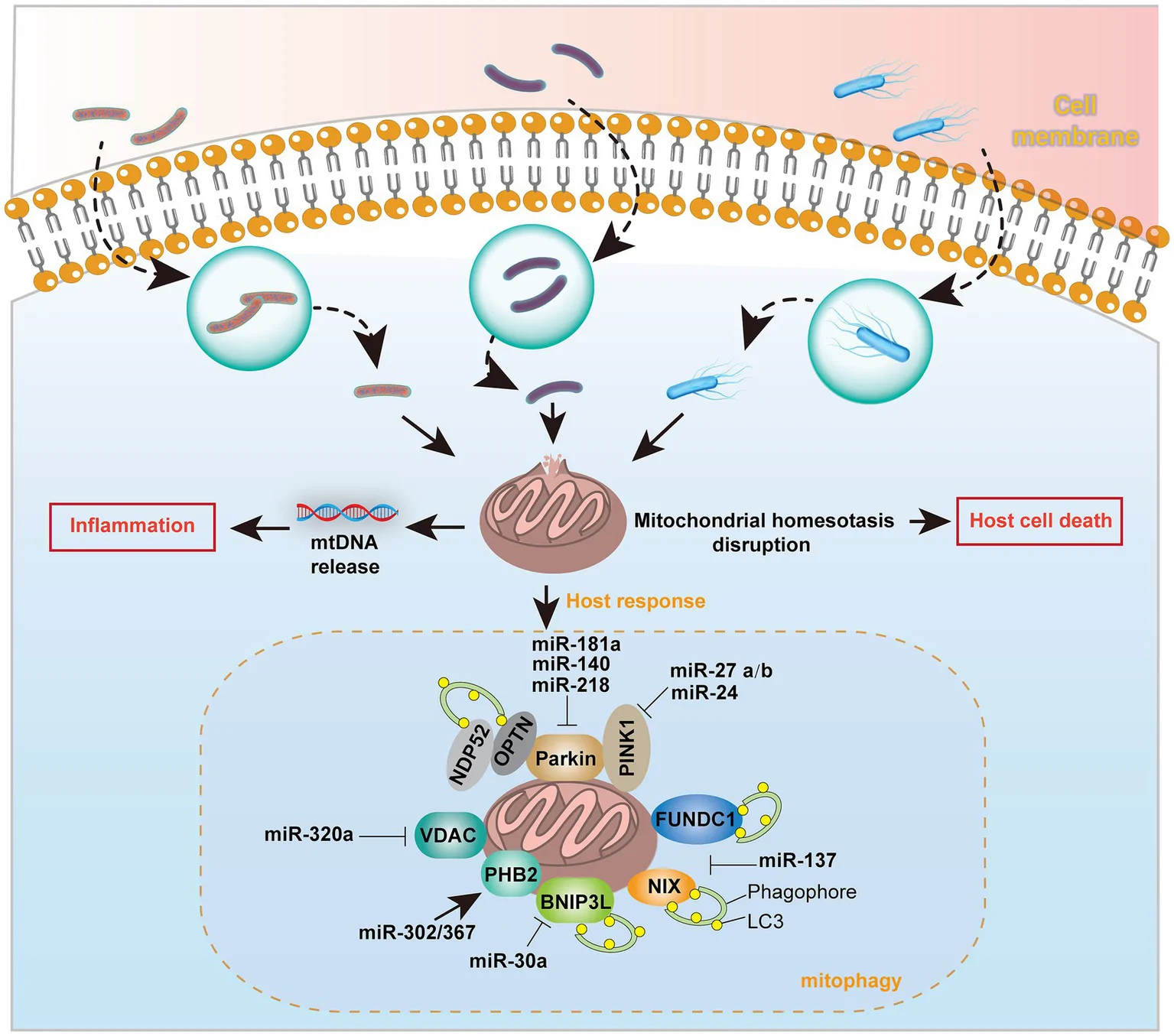
Overview of miRNAs regulating mammalian mitophagy pathways. Bacterial infections could cause mitochondrial damage and subsequent induces the cell death of mammalian cells or inflammatory response, favouring the bacterial survival or proliferation. While host cells activate a defense mitophagy responses through manipulating the expression of diverse miRNAs, and attenuates the host cell death and inflammation, which controlling the bacterial replication niche. See text for more details.

### 4.3 miRNA regulation of mitophagy in bacterial infection

Several correlations between miRNAs, mitophagy pathway and cancers have been identified in the last few years (Strappazzon, 2020), emphasizing the potential new strategies for cancer prevention or treatments (Rosolen et al., 2023). However, the roles of miRNA in regulating mitophagy for against bacterial infections remains largely unknown (Table 2).

**Table 2:** Interplay between microRNA and mitophagy.

miRNA	Mitophagy pathway	Target	Outcomes	Model system	Evidence level	References
miR-27a/miR-27b	PINK1-mediated	PINK1	Inhibition of mitophagy leading to mitochondrial damage	Non-infectious disease model	Direct experimental validation	Kim et al. (2016)
miR-181a	Parkin-mediated	Parkin	Suppression of Parkin-dependent mitophagy, triggering apoptosis	Non-infectious disease model	Direct experimental validation	Cheng et al. (2016)
miR-218	PINK1/PRKN-mediated	Parkin	Down-regulation of Parkin represses mitochondrial clearance	Non-infectious disease model	Direct experimental validation	Di Rita et al. (2020)
miR-140-5p	Parkin-dependent	Parkin	Modulation of Parkin mitochondrial translocation to impact mitophagy	Non-infectious disease model	Direct experimental validation	Zhang, Q. et al. (2022)
miR-24	PINK1/PRKN-mediated	PINK1	Modulation of PINK1 expression to maintain mitochondrial homeostasis	Non-infectious disease model	Direct experimental validation	Zhou et al. (2023)
miR-137	FUNDC1-mediated	FUNDC1/NIX	Targeting FUNDC1/NIX results in inhibited mitochondrial degradation	Non-infectious disease model	Direct experimental validation	Li et al. (2014)
miR-145	Bnip3-mediated	Bnip3	Repression of Bnip3 expression leads to inhibited mitophagy	Non-infectious disease model	Direct experimental validation	Liu et al. (2019)
miR-126-5p	Bnip3-mediated	PGC1α	Suppression of Bnip3-related mitophagy contributes to apoptotic death of chondrocytes	Non-infectious disease model	Direct experimental validation	Kim et al. (2021)
miR-30a-5p	BNIP3L/NIX-Dependent	Bnip3L	Targeted repression of Bnip3L alters mitochondrial metabolic activity	Non-infectious disease model	Direct experimental validation	Chevalier et al. (2022)
miR-let-7e	NIX-Dependent	IGF-1	Targeting IGF-1 to negatively or positively regulate NIX-dependent mitophagy	Non-infectious disease model	Direct experimental validation	Dabin et al. (2022)
miR-24-3p	FUNDC1-mediated	PHB2	PHB2-dependent mitophagy modulation contributes to reduction of cardiac fibrosis	Non-infectious disease model	Direct experimental validation	Zhang, Y. et al. (2022)
miR-302/367	PHB2-mediated	NFκB	PHB2-associated mitophagy-NFκB axis promotes bacterial clearance	Bacterial infection mode	Direct experimental validation	Huang et al. (2020b)
miR-218-5p	Parkin-mediated	Parkin	Down-regulation of Parkin causes inhibited mitophagy	Non-infectious disease model	Direct experimental validation	Naso et al. (2024)
miR-182/miR-326	Parkin-mediated	FOXP3	Targeting FOXP3 for negative regulation of Parkin-dependent mitophagy	Non-infectious disease model	Direct experimental validation	Zhang et al. (2024)

Previous study has confirmed that a novel miR-302b can regulate the production of proinflammatory cytokines in macrophages in response to Gram-negative bacterial infections, indicating that miR-302s may be crucial in macrophage-mediated immune defense (Zhou et al., 2014). Our recent research showed that the expression of miR-302/367 cluster was increased in the process of *P. aeruginosa* infection (Huang et al., 2020b). In macrophages, overexpression of miR-302/367 cluster enhanced the mitophagic responses, reduced ROS and bacterial elimination. Moreover, miR-302/367 cluster promoted bacterial clearance in a mouse infection model. Importantly, microRNA302/367 clusters regulate host mitophagy by targeting NF-κB to respond to *P. aeruginosa* invasion, promote the elimination of intracellular *P. aeruginosa*, and thus maintain cell homeostasis. The findings demonstrated that miR-302/367 cluster might be a potential regulator in mitophagy-mediated *P. aeruginosa* elimination and reveal a novel link between miRNAs and mitophagy for combating bacterial infection.

In contrast, *M. tuberculosis* offers a bridging axis: miR-155 targets Rheb/mTOR to enhance autophagy and restrict bacterial growth, but direct regulation of PINK1/PRKN or receptor-mediated mitophagy remains less defined (Wang et al., 2013). Additionally, miR-27a is abundantly expressed in active TB patients and *M. tuberculosis*-infected mice, which could directly suppress PINK1 translation, thereby inhibiting PINK1/Parkin-dependent mitophagy, suggesting that miR-27a might play a key role in regulation of host mitophagy during *M. tuberculosis* infection (Liu et al., 2018; Zhao, F. et al., 2023). For *L. monocytogenes*, the pore-forming toxin LLO triggers calcium influx and mitochondrial damage, which activate the novel mitophagy receptor NLRX1 via LRR-domain release, promoting bacterial survival by reducing mtROS; notably, this pathway bypasses PINK1/Parkin and is not yet linked to specific miRNAs (Bi et al., 2024). Across pathogens, these interactions converge on shared nodes—PINK1/Parkin, PHB2, BNIP3L/NIX, FUNDC1—regulated by NF-κB, p53, mTOR, and mitochondrial dynamics. Outcomes are pathogen-specific: mitophagy is protective in *P. aeruginosa*, exploited by *L. monocytogenes* via NLRX1, and subverted by *M. tuberculosis* through effector-mediated flux changes.

The miRNA-regulated mitophagy shapes antibacterial immunity by controlling damaged mitochondria, mtROS, and mtDNA release. In *P. aeruginosa*, the miR-302/367 cluster inhibits NF-κB to promote PHB2-dependent mitophagy and reducing mtROS (Huang et al., 2020b). This limits activation of NLRC4/NLRP3 inflammasomes, cGAS–STING, and TLR9 signaling, thereby tempering excessive inflammation while supporting bacterial clearance (Wang et al., 2022; Wang, J. et al., 2025). Conversely, impaired mitophagy amplifies these innate pathways via accumulated mtDAMPs (Marchi et al., 2023). In *M. tuberculosis* and *L. monocytogenes*, mitophagy can be co-opted to suppress mtROS, facilitating pathogen persistence (Zhang et al., 2019). Thus, the miRNA–mitophagy axis sets the threshold for innate immune activation and host defense.

Notably, pathogens actively intervene in these processes. Secreted bacterial toxins and secretion system-delivered effectors can cause mitochondrial damage, directly interfere with mitophagy receptor/PINK1-PRKN pathway components, and reprogram host miRNA expression. In this scenario, changes in miRNA abundance and mitophagy are downstream consequences of bacterial virulence actions rather than host-initiated defense responses. Currently, only a limited number of effectors and corresponding miRNA-mitophagy alterations have been characterized, and many T3SS/T4SS substrates remain unstudied for their capacity to perturb this host regulatory module.

### 4.4 MiRNAs and mitophagy as therapeutic targets for bacterial infection

Given that miRNAs function as the crucial regulators in bacterial infections, it is suggested that miRNAs can be used as potential novel therapeutic targets for medical interventions. To date, approximate 10 miRNA drugs have been investigated in clinical trials for several diseases, including infectious diseases (Kimura et al., 2023; Nunes et al., 2025). As the first miRNA-based therapy drug, MRX34 has been under clinical testing in patients with primary liver cancer since 2013 (Bouchie, 2013). Miravirsen, a novel antisense oligonucleotide that binds and inhibits miR-122, leading to a significant reduction in HCV infection (Janssen et al., 2013). In addition, studies on miR-34a in tumors have contributed to the development of MRX34, a potent therapeutic candidate for myeloma (Beg et al., 2017). Although no miRNA-based drugs were used to treat bacterial infection in clinical research up to now (https://clinicaltrials.gov/), miRNAs still represent the promising agents for future therapies against those notorious pathogens. Several important miRNAs were exhibited excellent immune regulation efficiency, such as miR-155, miR-146a, miR-29, miR-302b, and miR-33 (Du et al., 2018; Ma et al., 2011; Ouimet et al., 2016; Zhou et al., 2014). Notably, miR-155 was found to be the most commonly dysregulated miRNA in various bacterial infections such as *H. pylori*, *L. monocytogenes*, *M. tuberculosis*, and *S. aureus* mentioned above, suggesting their great potential for further clinical studies.

In addition to miRNAs, mitophagy can be used as potential novel therapeutic targets for bacterial infections. Regarding drug development, thiopeptide antibiotics and imiquimod have been shown potent antibacterial activity against *M. tuberculosis* in macrophage infection model, and induced mitophagy as mentioned above (Bird et al., 2020; Gao et al., 2025; Lee et al., 2020). A recent study revealed that a novel MvfR inhibitor (D88) and mitochondrial-targeted peptide (Szeto-Schiller 31), alleviated the *P. aeruginosa*-caused mitochondrial disturbances by regulating mitophagy-related pathway in skeletal muscle (Aggarwal et al., 2024). Thus, targeting mitophagy-related pathway presents the potential therapeutic strategy for optimizing clinical outcomes in *P. aeruginosa*-infected patients. Although no chemical drugs were used to target mitophagy for antibacterial infection in clinical research to date (https://clinicaltrials.gov/), mitophagy still represent the promising candidate for future therapies against those important pathogens. Nevertheless, substantial translational barriers remain for miRNA-oriented host-directed therapeutics. Critical challenges include insufficient *in vivo* miRNA delivery efficiency, poor biological stability in biological fluids, potential off-target effects on unrelated gene networks, and risk of perturbing physiological basal mitochondrial homeostasis upon long-term manipulation of mitophagy (Wang, Z. et al., 2025). These bottlenecks severely restrict clinical translation and must be carefully considered before clinical application.

These pressing gaps, together with the integrated framework presented above, set the stage for the concluding section, in which we summarize the current state of the field, discuss the key translational barriers, and offer perspectives for future mechanistic and therapeutic investigation.

## 5 Summary and perspective

Bacterial infections cause millions of deaths in worldwide for every year. It is urgently necessary to develop alternative therapeutic strategies to combat the increasing occurrence of multidrug-resistant bacteria, not just depends on classical antibiotic therapy. Here, we reviewed the crucial roles of miRNAs and mitophagy during bacterial infections and might provide new potential strategies to control diseases caused by these important pathogens. During the past decade, numerous studies have validated the critical role of miRNAs in various types of diseases, such as tumor, virus infection and bacterial infections (Rupaimoole and Slack, 2017; Vishnoi and Rani, 2023; Zhou et al., 2018). Many convincing evidences have supported their regulatory role in mitophagy, which provide a comprehensive understanding of molecular mechanisms linking miRNA deregulation to the mitophagic process (Cheng et al., 2016; Di Rita et al., 2020; Kim et al., 2016; Zhang, Q. et al., 2022; Zhou et al., 2023). Since the gene products that participate in mitophagy is continually identifying (Chevalier et al., 2022; Dabin et al., 2022; Kim et al., 2021; Liu et al., 2019; Zhang, Y. et al., 2022), it is possible that other important mitophagy-associated miRNAs will be discovered in the future (Naso et al., 2024; Wang et al., 2024; Zhang et al., 2024).

Moreover, since the multifariousness of bacterial infections, one miRNA may manipulate several targets of mitophagy pathway in different stages throughout the process of bacterial infection. The precise mechanism regarding the underlying interplay between miRNAs and diverse mitophagy-related genes is necessary to be investigated in the process of infection. Notably, it is also critical to develop more efficient tools for identifying novel miRNAs and manipulating mitophagy, since miRNAs and mitophagy could act as therapeutic targets for bacterial infections at different molecular levels. Altogether, such efforts are imperative to promote the comprehensive understanding of various miRNAs in infectious diseases by modifying the mitophagy process and will in turn provide new therapeutic strategies to combat and control infectious diseases.

Key unresolved scientific questions remain in this research field: (1) Which miRNA-mitophagy axes exert pathogen-specific effects versus broad-spectrum host responses against bacterial infection? (2) How do bacterial toxins and secretion systems reshape miRNA-mitophagy circuits in distinct host cell populations? (3) How can mitophagy be pharmacologically tuned without disrupting the basal mitochondrial homeostasis? Emerging approaches, including single-cell sequencing, spatial transcriptomics, multi-omics integration, genome-wide CRISPR screening, and advanced mitophagy reporters, will be essential to dissect these mechanisms at high resolution. Beyond basic science, targeting the miRNA-mitophagy axis holds promise as a host-directed therapy to boost antibacterial immunity or limit immunopathology. However, clinical translation faces significant challenges as mentioned above, including efficient miRNA delivery, systemic stability, off-target effects, and the risk of disrupting normal mitochondrial homeostasis. Overcoming these barriers will require interdisciplinary efforts combining microbiology, immunology, and drug-delivery technologies.
